# CANNABIS USE AND RISK OF FALLING IN OLDER ADULTS

**DOI:** 10.1093/geroni/igac059.395

**Published:** 2022-12-20

**Authors:** Thorsten Rudroff

**Affiliations:** University of Iowa, Iowa City, Iowa, United States

## Abstract

The prevalence of cannabis use has significantly increased among US adults ≥ 50 years. However, the effect of chronic cannabis use on fall risk in older adults is unclear. A series of investigations were conducted to examine the intersection between cannabis use and fall risk in older adults. The findings indicated that: 1) Older (≥ 50 years) chronic cannabis users have a higher fall risk and walking impairments, 2) cannabis users might have a discrepancy between perceptual and physiological fall risk, and 3) chronic use of Δ-9-tetrahydrocannabinol (THC) might have negative influences on inhibitory control and brain activity. Future mechanistic (e.g., neuroimaging) investigations of the short- and long-term effects a variety of cannabis products (e.g., THC/CBD ratios, routes of administration) on cognitive/motor function, and fall incidence in older adults are suggested.

